# Efficacy of Olyset® Plus, a New Long-Lasting Insecticidal Net Incorporating Permethrin and Piperonil-Butoxide against Multi-Resistant Malaria Vectors

**DOI:** 10.1371/journal.pone.0075134

**Published:** 2013-10-08

**Authors:** Cédric Pennetier, Aziz Bouraima, Fabrice Chandre, Michael Piameu, Josiane Etang, Marie Rossignol, Ibrahim Sidick, Barnabas Zogo, Marie-Noëlle Lacroix, Rajpal Yadav, Olivier Pigeon, Vincent Corbel1

**Affiliations:** 1 Institut de Recherche pour le Développement (IRD), Maladies Infectieuses et Vecteurs, Ecologie, Genetique, Evolution et Controle (MIVEGEC), UM1-UM2-CNRS 5290 IRD 224, Cotonou, Bénin; 2 Centre de Recherche Entomologique de Cotonou (CREC), Cotonou, Bénin; 3 Institut de Recherche pour le Développement (IRD), Maladies Infectieuses et Vecteurs, Ecologie, Genetique, Evolution et Controle (MIVEGEC), UM1-UM2-CNRS 5290 IRD 224, Montpellier, France; 4 Laboratory of Medical Entomology, Organisation de Coordination pour la lutte contre les Endémies en Afrique Centrale (OCEAC), Yaoundé, Cameroon; 5 Centre Supérieur des Sciences de la Santé, Université Catholique d’Afrique Centrale, Yaoundé, Cameroon; 6 Faculty of Medicine and Pharmaceutical Sciences, The University of Douala, Douala, Cameroon; 7 Vector Ecology and Management, Department of Control of Neglected Tropical Diseases, World Health Organization, Geneva, Switzerland; 8 Walloon Agricultural Research Centre (CRA-W), Agriculture and Natural Environment Department, Plant Protection Products and Biocides Physico-chemistry and Residues Unit, Gembloux, Belgium; 9 Department of Entomology, Faculty of Agriculture, Kasetsart University, Bangkok, Thailand; University of Crete, Greece

## Abstract

Due to the rapid extension of pyrethroid resistance in malaria vectors worldwide, manufacturers are developing new vector control tools including insecticide mixtures containing at least two active ingredients with different mode of action as part of insecticide resistance management. Olyset® Plus is a new long-lasting insecticidal net (LLIN) incorporating permethrin and a synergist, piperonyl butoxide (PBO), into its fibres in order to counteract metabolic-based pyrethroid resistance of mosquitoes. In this study, we evaluated the efficacy of Olyset® Plus both in laboratory and field against susceptible and multi-resistant malaria vectors and compared with Olyset Net, which is a permethrin incorporated into polyethylene net. In laboratory, Olyset® Plus performed better than Olyset® Net against susceptible *Anopheles gambiae* strain with a 2-day regeneration time owing to an improved permethrin bleeding rate with the new incorporation technology. It also performed better than Olyset® Net against multiple resistant populations of *An. gambiae* in experimental hut trials in West Africa. Moreover, the present study showed evidence for a benefit of incorporating a synergist, PBO, with a pyrethroid insecticide into mosquito netting. These results need to be further validated in a large-scale field trial to assess the durability and acceptability of this new tool for malaria vector control.

## Introduction

The recent decline of malaria burden relies largely on the massive use of insecticide treated bed nets (ITNs) and the artemisinin combination therapy (ACT), supported by indoor residual spraying of insecticides (IRS) and intermittent preventive treatment during pregnancy [1]. Nevertheless malaria is still a major public health issue with an estimated 655,000 deaths a year mostly among young children and pregnant women in sub Saharan Africa [1]. Resistance mechanisms of malaria parasites to antimalarial drugs and *Anopheles* vectors to insecticides are challenging the efficacy of malaria control tools [2]–[4].

Indeed the efficacy of ITNs and long-lasting insecticidal nets (LLINs) so far relies exclusively on a single class of insecticides, the pyrethroids, to which various resistance mechanisms have spread among malaria vector populations [5]–[7]. To date, four types of resistance mechanisms against insecticides have been described: metabolic resistance, target site resistance, penetration resistance and behavioral resistance [8].

Metabolic resistance involves the sequestration, metabolism and/or detoxification of the insecticide, largely through the overproduction or increased activity of specific groups of enzymes [9], [10]. Three main groups of enzymes have been identified: carboxylesterases, glutathione-S-transferases or GSTs and cytochrome P450-dependent monoxygenases. All these enzyme groups are involved in the resistance to pyrethroids used for malaria vector control [7].

Target site resistance is achieved by point mutations that render the actual targets of an insecticide less sensitive to the active ingredient [7], [11]. Pyrethroids are neurotoxic compounds targeting the sodium channels (responsible for raising the action potential in the neurons during the nerve impulses). Two well know mutations (1014S and 1014F) in the gene sequence of this sodium channel that confer resistance to pyrethroid insecticides are spreading across vector populations. However, an additional substitution (N1575Y) has been recently identified as a new genetic marker of pyrethroid resistance in African malaria vectors [12]. Penetration and behavioural resistance mechanisms are less documented nevertheless their role in the phenotypic resistance might be crucial [13], [14]. Clearly much more work is required in order to identify the significance of cuticular resistance in insecticide resistance.

Recent studies reported the loss of insecticidal efficacy [2] and protective effect of pyrethroid-treated bed nets [3] in Benin, highlighting the urgent need for alternative strategies to fight against resistant mosquitoes. This trend is not specific to the multi-resistant *Anopheles gambiae* populations in southern Benin alone [15]. Indeed a multi-centre study confirmed that deltamethrin coated LLIN did not kill as many mosquitoes in areas with metabolic resistance or target site mutation resistance as in susceptible areas [16].

In this context, scientists and manufacturers are working closely in order to propose new tools and strategies to fight efficiently against resistant malaria vectors. Among the new vector control tools is Olyset® Plus LLIN made of polyethylene netting incorporating permethrin as insecticide and piperonyl butoxide (PBO) as a synergist. This net is now available for use in public health. Permanet 3.0®, a combination LLIN with both deltamethrin and PBO incorporated in the top panel of the net has been widely evaluated in different ecological settings and showed equal or better performances than PermaNet® 2.0 against pyrethroid-resistant *An. gambiae s.l.* populations [16]–[19]. However the deltamethrin content on PermaNet® 3.0 is up to twice as much as that of PermaNet® 2.0. This feature impeded any conclusion about the additive effect of the PBO on the top panel of PermaNet® 3.0. Nevertheless mathematical modeling showed that PermaNet® 3.0 might provide better community level protection than PermaNet® 2.0 in areas of pyrethroid resistance especially when the LLINs are extensively washed and/or torn [20].

The Olyset® Plus manufactured by Sumitomo Chemical is a 150 denier net containing a similar dose of permethrin (2% w/w) as Olyset Net® [21], but in addition has PBO (10 g/kg) in the whole net. Both permethrin and PBO are incorporated into the polyethylene fibres. The technology to incorporate these chemicals into the netting fibres is slightly different than the one used for Olyset Net® leading to an enhanced bleeding rate with a regeneration time of one day [22]. The current paper presents results of evaluation of the performance of this new generation LLIN in laboratory and semi-field conditions against both susceptible and resistant *Anopheles gambiae* strains in experimental huts in Benin and Cameroon. Standard World Health Organization (WHO) procedures were followed to investigate the regeneration time, washing resistance and efficacy of Olyset Plus in terms of induced exophily, blood-feeding inhibition and mortality in comparison with the standard Olyset Net [23].

## Materials and Methods

### Biological Material

Non-blood fed, 2–5 days old females of susceptible *Anopheles gambiae s.s.* (Kisumu strain) were used for the evaluation of efficacy in the laboratory and for release-recapture experiments. The Kisumu strain is fully susceptible to insecticides and free of any detectable insecticide resistance mechanisms; it originated from Kenya and has been colonized for many years in the laboratory. The susceptibility of this mosquito strain to insecticides is checked every 3 months using PCR. The pyrethroid resistant mosquitoes were bred in the laboratory from the larvae collected from natural breeding sites in Cotonou (6°21N-2°23E), Benin and Pitoa (9°21N; 13°31E), Cameroon.

### Regeneration and Wash Resistance Studies

To determine the regeneration time of the LLINs after standard washing and holding at 30°C, bioassays were carried out at constant intervals of time (+1, +2, +3, +5, +7 days) on 6 net samples (4 Olyset® Plus and 2 Olyset Net®) washed and dried three times consecutively following WHO procedures [23]. Details of standard washing and bioassays are provided in the WHO guidelines for testing and evaluation of LN [24]. Insecticide bioavailability curves (of 24 hour mortality and 60 min KD), as measured by 3 minutes exposure in cone bioassays, were established before washing 6 samples and after washing them three times consecutively on a day, and tested within a maximum of 7 days post-washing. The time required (in days) to reach initial level or a “plateau” is the period required for full regeneration of Olyset® Plus, i.e. RT value as per WHO guideline [24]. Regeneration time studies were supplemented by determination of the median knock down time as described by Skovmand et al. [25] in order to measure the dynamics of the insecticide after washing. In this study, bioassays were carried out at constant intervals of time (+1, +2, +3, +5, +7 days) on 6 net samples (4 Olyset® Plus and 2 Olyset Net®) washed and dried three times consecutively using the circular chamber. The subsequent mortality rates were then compared with those obtained through WHO cone test.

The wash resistance of Olyset® Plus was determined in standard bioassays on nets washed at the intervals equivalent to the regeneration time as described above. Standard WHO washing procedure was used. Netting samples were dried and held at 30°C between the subsequent washes and cone bioassays were performed after 1, 3, 5, 10, 15, 20 and 25 washes [23]. Each bioassay was done after the elapse of regeneration time but just before the next wash.

### Bioassay Procedures

#### WHO cone test method

The WHO cone test measures knock down and mortality of mosquitoes exposed in a small chamber to a piece of treated netting for a 3 min exposure time [23]. Five, non-blood fed, 2–5 days old *An. gambiae* mosquitoes were exposed for 3 min to netting pieces cut from five positions and held for 24 h with access to sugar solution. Fifty mosquitoes (5 mosquitoes × 10 cones) on each of the and 6 netting samples (25×25 cm) were tested and results pooled for analysis. Mosquitoes exposed to untreated nets as well as to permethrin conventionally treated nets (500 mg AI/m^2^) were used as a negative and positive control, respectively. Bioassays were carried out at 27±2°C and 75±10% RH. Knock down was measured after 60 min of exposure and mortality after 24 h.

#### Circular chamber test method

The measure of median knock-down time (MKDT) i.e. time at which 50% mosquitoes are knocked down allowed us to follow the variations of insecticide surface concentration in treated materials [25]. Batches of eleven non-blood fed, 2–5 days old, *An. gambiae* mosquitoes were introduced into a circular chamber, 10 cm diameter and 1 cm height that forces a complete contact with netting material. The time to knock-down (in seconds) for each individual mosquito was recorded. The MKDT was read off from the list (corresponding to the time of knock-down of the sixth mosquito in a sample of eleven mosquitoes). Four replicates of eleven mosquitoes were used for each netting sample. Six netting samples (4 Olyset® Plus and 2 Olyset Net) were tested and results pooled for analysis.

### Experimental Hut Trials

#### Design of the huts

The huts are made from concrete bricks, with a corrugated iron roof, a ceiling of thick polyethylene sheeting, and a concrete base surrounded by a water-filled channel to prevent entry of ants [26]. Mosquito access is *via* 4 window slits constructed from pieces of metal, fixed at an angle to create an inverted funnel with a 1 cm wide gap. Mosquitoes fly upward to enter through the gap and downwards to exit; this precludes or greatly limits exodus though the aperture enabling the majority of entering mosquitoes to be accounted for. A single veranda trap made of polyethylene sheeting and screening mesh (measuring 2 m long, 1.5 m wide, and 1.5 m high) projects from the back wall of each hut. Movement of mosquitoes between hut and veranda is unimpeded during the night.

#### Study areas

The release-recapture studies were conducted in 2 experimental stations belonging to the Anopheles Biology & Control (ABC) network. *An. gambiae s.l*. populations at each site present a different pattern of pyrethroid-resistance.

Pitoa (9°21N; 13°31E) is a small village with about 5,000 inhabitants, located at 15 km from Garoua in an area of extensive cotton cultivation in Northern Cameroon (about 35 000 ha cultivated area). *An. gambiae s.l.* and *An. funestus* s.l. are the main malaria vectors in this area. *An. arabiensis* is predominant and previously showed moderate level of resistance to permethrin, deltamethrin and DDT [27] due to higher oxidase and esterase activities [28], [29]. Only *An. arabiensis* mosquitoes were released into the huts for the release-recapture experiment.

Akron (6°30′N; 2°47′E) is located in the district of Porto-Novo, the capital of Benin (coastal Guinean area). The experimental hut stations are located in the outskirts of the city, near the Lake Nokoué close to a vegetable farm of 20 hectares. In this study, *An. gambiae* s.s. (100% M molecular form) from Cotonou (and not from Akron) were released into the huts. *An. gambiae* from Cotonou shows similar resistance pattern similar to Akron with very strong resistance to permethrin (<20% mortality when exposed to the diagnostic dose); 1014F *kdr* frequency is high (0.90) and metabolic resistance through increased oxidase activity has also been reported [30], [31].

The experimental hut trial with wild mosquito population was carried out in Malanville (11°87N; 03°38E), a “sous-préfecture” of north-Benin, located in a soudanian savannah area, near rice fields. The area is characterized by a long dry season lasting from December to June. An irrigation system from the Niger river allows practicing rice cultivation during the dry season. *An*. *gambiae s*.*l*. is the main malaria vector, with 95% *An*. *gambiae s*.s, M form, and 5% *An*. *arabiensis*. Increased resistance of malaria vectors to pyrethroids (22% mortality with 0.75% permethrin test papers in 2010) was recently reported in Malanville due to increased oxidase activity and high prevalence of the 1014F *Kdr* mutation (freq*_Kdr_* = 0.5 in 2010) [30].

#### Release-recapture experiment

The efficacy of Olyset® Plus was evaluated against susceptible (Kisumu) and pyrethroid resistant strains (Pitoa and Cotonou) of *An. gambiae* released in separate experimental huts early in the evening and re-captured the next morning.

The following comparison arms were tested in separate huts:

Olyset Net® (permethrin incorporated into polyethylene net)
Olyset® Plus without PBO (only permethrin incorporated into polyethylene net)
Olyset® Plus (permethrin+PBO incorporated into polyethylene net)
Untreated polyethylene net (negative control)

Olyset® Net appeared identical to Olyset® Plus without PBO and permethrin although their production technologies were completely different. Olyset® Plus without PBO used the same formulation technology and bleed rate as that of Olyset Plus except that PBO was not incorporated into it. This therefore allowed us to directly investigate the impact of PBO by comparing the performance of Olyset Plus with and without PBO.

Before testing in the experimental huts, the nets (including control) were deliberately holed (6 holes of 4 cm×4 cm) to simulate torn nets according to the WHO procedure [24].

#### Procedure of experimental hut trial

The field trial of Olyset® Plus was carried out in Malanville, Benin in collaboration with the WHO Pesticide Evaluation Scheme (WHOPES). Washed and unwashed Olyset® Plus and Olyset® Net were evaluated in experimental huts on free-flying, wild An. gambiae mosquitoes for their effects to deter mosquito entry, repel or drive them out of huts, induce mortality and inhibit human blood-feeding.

The following comparison arms were tested:

1. Unwashed Olyset® Plus
2. Olyset® Plus washed 20 times
3. Unwashed Olyset® Net
4. Olyset Net® washed 20 times
5. Polyester net conventionally treated with permethrin (500 mg active ingredient (AI)/m^2^) and washed to just before exhaustion (positive control).
6. Untreated polyester net (negative control)

The polyester nets were conventionally treated with permethrin emulsifiable concentrate at 500 mg AI/m^2^ dose at Centre de Rechercehe Entomologique de Cotonou (CREC), Benin. For wash resistance, the nets were washed at CREC according to a protocol adapted from the standard WHO washing procedure [24] and at the interval equal to the regeneration time previously established in the laboratory for each LLIN. The point of exhaustion for conventionally treated nets was determined by washing the conventionally treated nets using the Phase II protocol according to WHOPES procedures [23]. Nets were dried horizontally in the shade and stored at ambient temperature between the washes. WHO cone bioassays were performed just before the subsequent wash. The last wash after which the net still caused >80% mortality or >95% KD was considered to be the number of washes required before exhaustion.

Before testing in the experimental huts, the nets including the control nets were deliberately holed I.e. 6 holes measuring 4 cm×4 cm were made in each net, 2 holes in each of the long side panels, and one hole at each end (head- and foot-side panels).

Each week, the treatment arms were rotated among the huts according to a Latin square scheme. Six nets were used per treatment arm and each of the 6 nets were tested one night during the week. At the end of the week, the huts were properly cleaned and ventilated to remove potential contamination. The treatment was then rotated to a different hut. The trial lasted for 12 weeks to ensure two complete Latin square rotations through the huts which were necessary to obtain sufficient numbers of mosquitoes for statistical analysis.

### Bioassays

Six nets (one per treatment arm) were bio-assayed at time 0 (i.e. the day before the first wash). Bioassays were carried out for a second time after completing necessary number of washes and then for a third time at the end of the field trial with the nets that were used in the huts using the susceptible Kisumu strain according to the WHO procedures for cone test. Bioassays were replicated five times to ensure that 50 mosquitoes in overall were tested per net. Knock down was checked 60 min after exposure and mortality 24 h after exposure. For the conventionally treated nets, bioassays were done before and after each wash and the washing stopped just before the cut-off point (i.e. the last wash for which the net still causes >80% mortality or >95% KD).

A 7^th^ net in each of the six comparison arms was not tested in the huts and kept for chemical assays. Before wash one piece of 30 cm×30 cm netting was taken from each of the 5 panels of the 6 nets according to the WHO sampling procedure and kept for chemical analysis. After washing of nets and at the end of the hut trial, 5 pieces of netting were taken again from 5 adjacent positions of each net (including the unwashed nets as they lasted at least 6 weeks unused). These were put in labelled aluminium foils and kept in sealed bags and sent to the WHO Collaborating Centre for Quality Control of Pesticides, Gembloux, Belgium for chemical analysis. The 5 netting samples from each net were pooled and analyzed to provide the average insecticide content of the net and between- and within-net variation and density of netting.

### Chemical Analysis Protocol

For the wash resistance study, chemical analyses were performed on Olyset® Plus samples washed 0, 1, 3, 5, 10, 15, 20 and 25 times. After each wash cycle, 4 pieces (25 cm×25 cm) from 4 nets were analysed to determine the content of permethrin and piperonyl butoxide. For the Phase II trial, chemical analysis was performed on all unwashed and washed nets before and after the hut trial. Five pieces (25 cm×25 cm) were cut from each net according to the WHO sampling method for LLINs and pooled for chemical analysis. The average permethrin and PBO contents were determined using the CIPAC method 331/LN/M/3 (www.cipac.org). This method involved extraction of permethrin and PBO from the net samples in a water bath (85–90°C) for 45 minutes with heptane in the presence of triphenyl phosphate as internal standard and determination by gas chromatography with flame ionization detection.

### Mosquito Collection Procedures

Adult male or female volunteers slept under the nets in the assigned huts and were rotated randomly among huts each night of the study. They entered the hut at dusk and remained inside until dawn. Mosquitoes were collected in the morning. Dead mosquitoes were first collected off the floor, inside the nets and in the hut and the exit traps. Alive resting mosquitoes were collected individually using Pyrex tubes from inside the net and from the walls and roof of the hut and exit traps. Mosquitoes were scored by location as dead or alive and as blood-fed or unfed. Alive mosquitoes were placed in small cups and were provided with access to sugar solution for assessing delayed mortality after 24 h of holding.

The primary outcomes measured in experimental huts were:

deterrency (i.e. reduction in the number of mosquitoes relative to the control hut);
induced exophily (i.e. the proportion of mosquitoes that exited early and were found in the veranda trap relative to the control hut);
blood-feeding inhibition (i.e. reduction in the proportion of mosquitoes with blood-feeding relative to the control hut);
immediate and delayed mortality (i.e. the proportion of mosquitoes found dead in the morning and those that died 24 h of holding, respectively).

### Reporting of Adverse Events and Provision of Medical Care

Before the study, all sleepers underwent a medical check up. The sleepers in the huts were also asked during the study about perceived adverse or beneficial effects of each treatment. The volunteers were asked to report any adverse events associated with use of nets and a provision for medical care was made. The protocol was approved by the National Committee of Ethics of Benin (IRB00006860).

### Statistical Analysis

Data from cone bioassays were compared between each net using a Chi square test. Significance between treatments was set at 5% level. Data were analyzed using the Minitab Software Version 12.2. The number of mosquitoes of each species entering the huts was compared and analysed using the non-parametric Kruskal-Wallis test. The proportion of mosquitoes that exited early (induced exophily), the proportion that was killed within the hut (mortality) and the proportion that successfully blood-fed (blood-feeding rate) were compared and analysed using the logistic regression (XLSTAT Software version 2011).

### Ethical Considerations

Volunteers from the study villages were recruited after obtaining informed written consent. A medical doctor was on hand during the trial to respond to any side effects of the ITNs or to treat any cases of fever. Confirmed *P. falciparum* parasitaemia was treated with Coartem (artemether 20 mg/lumefantrine 120 mg). The protocol received approval from the national ethics committee of Benin and the Ministry of Health Review Board in Cameroon.

## Results

### Regeneration Time

**Figure 1:**
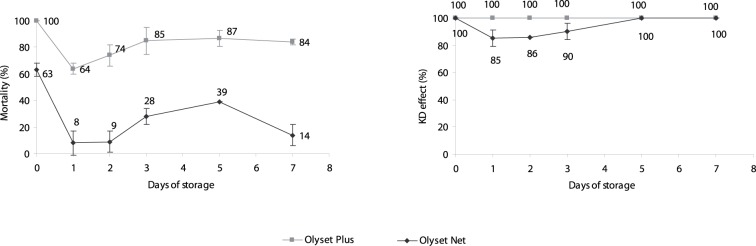
Mortality and knock-down (KD) effect at 60 min induced by Olyset® Plus and Olyset Net® unwashed and washed 3 times and after several days of storage.

The regeneration curve for Olyset® Plus and Olyset® Net is presented in Figure 1 where each dot represents a mean of ten replicates of 4 Olyset® Plus net samples and 2 Olyset Net® net samples, respectively.

Regardless the days of storage the KD effect was 100% for both unwashed and three times washed Olyset® Plus. The mortality was 100% for unwashed Olyset® Plus net but after three consecutive washes on same day the mortality decreased to 64%. Mortality then increased up to 87% after 5 days of storage of the net. The bioefficacy of Olyset® Plus LN never reached the initial level of mortality after washing but it reached a plateau between 3 and 7 days. There were no significant differences between mortalities induced 3, 5 or 7 days after the 3 washes (Pair wise comparison, Chi^2^, p>0.05). Moreover the difference between the mortality induced at 2 days after washes and 3 days after washes was also not significantly different (Pair wise comparison Chi^2^, p = 0.051). Based on these data, the regeneration time for Olyset® Plus was considered as 2 days.

The bioefficacy of the Olyset Net® was significantly lower than that of Olyset® Plus. Indeed, the KD effect was 100% for unwashed net but decreased to 85% after the first wash. Then, the KD of Olyset Net increased on storage reaching to 100% after 5 days of storage. Initial mortality was 64% for unwashed Olyset® Net. After 3 consecutive washes on same day, mosquito mortality decreased to less than 10%. Mortality increased up to 39% after 5 days of storage but decreased again after 7 days. There was no clear trend for a plateau of mortality with Olyset® Net in contrast to Olyset® Plus.

The permethrin content of Olyset® Plus decreased from 19.21±1.5 g AI/Kg to 15.95±1.0 g AI/Kg after 3 washes. In contrast, no significant decrease in permethrin content was noted with Olyset® Net (20.27±0 g AI/Kg before wash versus 19.77±1.0 gAI/Kg after 3 consecutive washes).

### Insecticide Dynamics on the Net Surface after Washing

**Figure 2:**
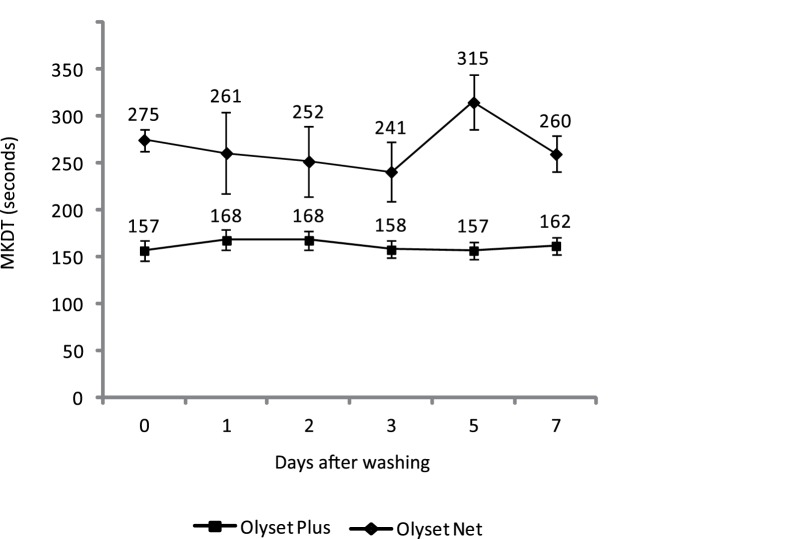
Average values of median knock-down time (MKDT) in seconds (±CI_95_) for samples unwashed and washed 3 times and after several days of storage.

The dynamics of permethrin in the fibres of Olyset® Plus and Olyset Net® was tested using circular chamber tests at different intervals of time (+1, +2, +3, +5, +7 days) after 3 consecutive washes. Figure 2 illustrates the values of the MKDT for unwashed and washed net samples. Each data point represents a mean of four replicates of 4 samples of Olyset® Plus and 2 samples of Olyset Net®, respectively.

For Olyset® Plus, no significant differences were observed between the average values of MKDT between unwashed and washed samples, regardless the days of storage. Conversely, the MKDT values for Olyset Net® were always above those of Olyset® Plus, indicating that longer time is needed to knock-down 50% mosquitoes with the reference Olyset Net. These data confirmed the lower bioavailability of permethrin on Olyset Net® which was expressed by lower mortality rate in cone tests.

### Wash Resistance of Olyset® Plus

Table 1 shows the efficacy of Olyset® Plus in terms of KD effect and mortality after 1, 3, 5, 10, 15, 20 and 25 washes. Each result represents a mean of 4 replicates. Olyset® Plus gave >95% KD which was above the WHO cut off point until 25 washes, whereas mortality decreased to 76% after third wash, which was below the WHO cut off after 3 washes.

**Table 1:** Average values of knock-down (KD) effect after 60 min and mortality at 24 h (±CI_95_) and active ingredient contents (g/Kg±SE) of Olyset® Plus sample after washes.

	Number of washes						
	1	3	5	10	15	20	25
KD effect (%±SE)	100	100	99.5±1	98.5±1	99±1.1	95.4±5	95.4±2.7
Mortality (%±SE)	100	75.9±8.3	42.9±8.6	21.5±6.8	35.6±8.5	15.3±7	15.8±9.5
Permethrin content (g/Kg ±SE)	18.10±0.29	16.05±0.22	14.86±0.19	13.87±0.39	13.01±0.27	12.32±0.34	11.38±0.43
PBO content (g/Kg ±SE)	7.39±0.21	6.47±0.12	5.84±0.11	5.16±0.29	4.60±0.17	4.00±0.28	3.79±0.22

The permethrin content in the unwashed Olyset® Plus complied with the target dose of 19.27±0.29 g AI/kg. The variation among Olyset plus® samples from the same net (i.e. the standard errors presented in Table 1) was low indicating a good homogeneity of the active substance’s distribution over the Olyset® Plus.

The variation of permethrin content between Olyset® Plus samples washed 1 to 25 times remained low. The average permethrin content was 16.1 g AI/kg after 3 washes (lower than for Olyset® Net, which was 19.8 g/kg after 3 washes), 13.9 g AI/kg after 10 washes and 12.3 g AI/kg after 20 washes. The overall permethrin retention in Olyset® Plus after 20 washes was 64.1%, corresponding to an average retention per wash of 97.8%.

The PBO content in the unwashed Olyset® Plus complied with the target dose of 10±2.5 g PBO/kg and the between-net variation showed a good homogeneity of its distribution over the net. The between-net variation of PBO content on Olyset® Plus samples washed 1 to 25 times remained low. The average PBO content decreased to 6.5 g PBO/kg after 3 washes, 5.2 g PBO/kg after 10 washes and 4.0 g PBO/kg after 20 washes. The overall retention of PBO after 20 washes was 44.2%, corresponding to an average retention per wash of 96.0%. (Table 1).

### Release-recapture

For the three *An. gambiae s.l.* strains used in release-recapture experiments, the mortality rates in the control huts were <5% and blood-feeding rates were >80%, indicating suitable environmental conditions for the tests.

With the susceptible (Kisumu) strain, all unwashed LLIN (Olyset Net®, Olyset® Plus without PBO or Olyset® Plus) induced >99% mortality and 100% blood-feeding inhibition (BFI) (Table 2). After 3 consecutive washes and 7 days storage, mortality rates (>93%) and BFI (>92%) for the three nets were still high (Table 2). Exophily rates of the LLINs ranging from 15 to 33% were significantly higher than in the control (Table 2).

**Table 2:** Summary of release-recapture experiments with susceptible and resistant populations of *Anopheles gambiae.*

	Net treatment	Washingregimen	Totalcollected	% caught inveranda (95% CI)	% blood-fed(95% CI)	% feedinginhibition	% mortality(95% CI)
***Anopheles gambiae*** ** Kisumu**							
Control		0	194	30 (23–36)^a^	84 (79–89)^a^	–	3 (0–5)^a^
		3	184	18 (12-23)^b^	84 (78–89)^a^	–	1 (0–2)^a^
Olyset Net		0	196	24 (18–30)^a^	0 (0–0)^b^	100	99 (98–100)^b^
		3	184	28 (22–35)^a^	3 (1–6)^b^	95	93 (89–97)^b^
Olyset Plus without PBO		0	184	22 (16–28)^a^	0 (0–0)^b^	100	100 (100–100)^b^
		3	169	31 (24–38)^a^	4 (1–7)^b^	95	99 (97–100)^b^
Olyset Plus		0	183	15 (10–21)^b^	0 (0–0)^b^	100	100 (100–100)^b^
		3	198	33 (27–40)^a^	0 (0–0)^b^	100	100 (100–100)^b^
***Anopheles gambiae*** ** Cotonou**							
Control		0	183	33 (26–40)^a^	77 (70–82)^a^	–	3 (0–5)^a^
		3	190	23 (17–29)^a^	86 (81–91)^a^	–	2 (0–3)^a^
Olyset Net		0	178	49 (42–56)^b^	12 (7–17)^b,d^	85	78 (72–84)^b^
		3	175	59 (52–67)^b^	26 (19–32)^c^	70	11 (7–16)^c^
Olyset Plus without PBO		0	169	59 (52–67)^b^	6 (2–9)^d^	92	80 (75–86)^b^
		3	181	73 (67–80)^b,c^	18 (12–23)^b,c^	80	18 (13–24)^c^
Olyset Plus		0	182	62 (54–69)^b^	14 (9–19)^b,c^	82	92 (88–96)^d^
		3	171	54 (47–62)^b,d^	11 (6–16)^b,d^	87	56 (48–63)^e^
***Anopheles gambiae*** ** Pitoa**							
Control		0	183	16 (11–21)^a^	85 (79–90)^a^	–	1 (0–2)^a^
		3	186	17 (11–22)^a^	81 (76–87)^a^	–	1 (0–2)^a^
Olyset Net		0	182	58 (51–65)^b,c^	2 (0–4)^b^	97	76 (70–82)^b^
		3	184	48 (41–55)^b^	10 (5–14)^c,d^	88	38 (31–45)^c^
Olyset Plus without PBO		0	164	45 (37–52)^b^	0 (0–0)^b^	100	96 (93–99)^d^
		3	189	51 (44–58)^b^	5 (2–8)^b,d^	93	52 (45–60)^e^
Olyset Plus		0	160	41 (34–49)^b,d^	0 (0–0)^b^	100	98 (95–100)^d^
		3	188	43 (36–49)^b,d^	3 (0–6)^b,d^	96	69 (62–75)^b^

For each *Anopheles gambiae* population, values in columns not sharing the same superscript letter are significantly different at the 5% level.

With the multi-resistant (kdr+metabolic) strains of *An. gambiae* from Cotonou, exophily (>49%), blood-feeding inhibition (>82%) and mortality (>77%) for all unwashed LLINs were significantly higher than the control (p<0.05). Olyset® Plus caused significantly higher mortality than Olyset Net® and Olyset® Plus without PBO (p<0.05), whereas the Olyset® Plus without PBO induced greater BFI than Olyset® Plus (P<0.05). After 3 washes (Table 2), significant decrease in mortality rates was observed with Olyset Net® (10%) and Olyset® Plus without PBO (17%), whereas that of Olyset® Plus remained high (55%). BFI remained high for the three LLIN (>70%) regardless the wash regimen. All LLINs significantly induced exophily (ranging from 43% to 73%) relative to the control (ranging from 23% to 33%) with the mutli-resistant mosquitoes from Cotonou regardless the wash regimen.

With the metabolic-based resistant strain of *An. gambiae* (from Pitoa), performance of unwashed LLIN was more or less similar to that found using the Kisumu strain, except for Olyset Net® that showed significantly lower mortality (76%) than the 2 other LLIN (97% and 96% respectively, p<0.05) (Table 2). BFI was high (>87%) regardless the LLIN and wash regimen (0 or 3). After 3 washes, however, overall mortality decreased for all LLINs but the lethal effect of the Olyset® Plus (68%) was significantly higher than that of Olyset Net® (37%) and Olyset® Plus without PBO (52%). All LLINs significantly induced exophily ranging from 41% to 58% relative to the control (16–17%) with the metabolic-based resistant mosquitoes from Pitoa regardless the wash regimen.

### Field Trial

LLINs were evaluated in the experimental huts of Malanville between 18^th^ September and 10^th^ December 2011, corresponding to 72 nights of collections per hut, i.e. 6 nights per week during 12 weeks (two complete Latin squares). All results of the chemical analysis and the entomological data are summarized respectively in Table 3 and 4 for *Anopheles gambiae* and other Culicidae.

**Table 3:** Active ingredient and synergist contents of Olyset® Net and Olyset® Plus net samples used in Phase II trial.

	Permethrin content (g/kg)			
Treatment	Before washing	After washing	AI retention(% of wash 0)	After testing
Olyset Net unwashed	19.68	19.95	–	19.69
Olyset Net 20 washes	19.62	16.72	85%	17.05
Olyset Plus unwashed	18.59	19.01	–	17.90
Olyset Plus 20 washes	18.59	14.46	78%	14.36
Polyester treated net	10.99	7.66	70%	3.90
Untreated net	<LD	<LD	–	<LD
	**Piperonyl butoxide content (g/kg)**			
**Treatment**	**Before washing**	**After washing**	**AI retention** **(% of wash 0)**	**After testing**
Olyset Net unwashed	<LD	<LD	–	<LD
Olyset Net 20 washes	<LD	<LD	–	<LD
Olyset Plus unwashed	8.73	8.96	–	8.12
Olyset Plus 20 washes	8.77	4.51	51%	4.25
Polyester treated net	<LD	<LD	–	<LD
Untreated net	<LD	<LD	–	<LD

<LD means below limits of detection.

**Table 4:** Summary of experimental hut trial results for *Anopheles gambiae* and the other *culicidae*.

	Net treatment	Totalcollected	% caught inveranda(95% CI)	Totalblood-fed	% blood-fed(95% CI)	% feedinginhibition	Totaldead	% mortality(95% CI)
***Anopheles gambiae***								
Control		69	22 (12–31)^a^	43	62 (51–76)^a^	–	0	0 (0–0)^a^
CTN before exhaustion		74	65 (54–76)^b,c^	12	16 (8–25)^b,c^	74	41	55 (44–67)^b,d^
Olyset Plus, unwashed		67	54 (42–66)^b^	7	10 (3–18)^b^	83	54	81 (71–90)^c^
Olyset Plus, 20 washes		101	53 (44–63)^b^	13	13 (6–19)^b^	79	68	67 (58–76)^b,c^
Olyset Net, unwashed		96	71 (62–80)^c^	11	11 (5–18)^b^	82	40	42 (32–52)^d,e^
Olyset Net, 20 washes		124	70 (62–78)^c^	31	25 (17–32)^c^	60	45	36 (28–45)^e^
**Other C** ***ulicidae***								
Control		821	34 (31–37)^a,d^	536	65 (62–69)^a^	–	12	1 (1–2)^a^
CTN before exhaustion		760	33 (30–37)^a,c,d^	20	3 (1–4)^b^	96	689	91 (89–93)^b^
Olyset Plus unwashed		613	29 (25–32)^b,c^	20	3 (2–5)^b,c^	95	590	96 (95–98)^c^
Olyset Plus, 20 washes		896	33 (30–36)^a,c^	29	3 (2–4)^b,c^	95	807	90 (88–92)^b^
Olyset Net, unwashed		662	38 (34–41)^d^	17	3 (1–4)^b^	96	580	88 (85–90)^b,d^
Olyset Net, 20 washes		805	37 (34–40)^a,d^	38	5 (3–6)^c^	93	686	85 (83–88)^d^

For each species, the numbers in the same column sharing the letter superscript do not differ significantly (p>0.05). CTN = conventionally treated net.

During the 72 nights of collections, 69 *An. gambiae s.l.* specimens were collected in the control hut (i.e. a mean number of 1 female caught per night). About 62% (43 of 69) of them were blood-fed. The “natural” exophily was 22% and mortality was nil. No significant reduction in mosquito entry rates (deterrency) was noted with all treatments compared to the untreated (control) arm. However, all treatments induced significantly higher exophily (from 140% to 225%) than the untreated net. High and significant blood-feeding inhibition (BFI) rates were observed with all LLIN treatments compared to the control (p<0.05). The BFI rate induced by Olyset® Plus washed 20 times (79%) was significantly higher than that of 20 times washed Olyset Net® (60%). All treatments killed significantly more mosquitoes (36%–81%) than none by the untreated net (p<0.05). The best insecticidal effect was obtained with the unwashed Olyset® Plus (81%) followed by Olyset® Plus washed 20 times (67%). To summarize, 20 times washed Olyset® Plus caused BFI and mortality rates similar to conventionally treated nets washed to just before exhaustion (74% and 55%, respectively).

During the 72 nights of collection, 821 Culicidae mosquitoes were collected in the control hut (i.e. a mean number of 11 females caught per night), 65% of them were blood-fed. The natural exophily was 34% and mortality was less than 2%.

No significant reduction in entry rates (deterrence) and exit rates (exophily) was noted with all treatments compared to the untreated (control) arm (p<0.05). High and significant BFI rates were observed with all treatments compared to the control (p<0.05). All LLINs (20 times washed and unwashed) induced BFI similar to that of the conventionally treated nets washed to just before exhaustion (95%) except for Olyset® Net washed 20 times that caused significantly lower BFI (92%). Similarly, all treatments induced strong killing effect (from 85% to 96%) compared to the untreated net (1.5%, p<0.05). The highest killing effect of 96% was induced by the unwashed Olyset® Plus.

The chemical analyses of the mosquito nets used for the field trial showed that the permethrin content of unwashed Olyset® Net and Olyset® Plus complied with the target dose of 20±3 g AI/kg and 20±5 g AI/kg respectively. In Olyset® Net and Olyset® Plus washed 20 times, it was 16.7 g AI/kg and 14.5 g AI/kg, respectively, corresponding to an overall retention of 85% and 78%, respectively. The differential permethrin AI load between Olyset® Plus and Olyset® Net was due to the difference in permethrin bleeding rate onto the net surface, which was found higher for Olyset® Plus than Olyset® Net. The PBO content in Olyset® Plus complied with the target dose of 10±2.5 g PBO/kg. It retained 4.5 g PBO/kg after 20 washes, corresponding to an overall retention of 51%. After the experimental hut study, the permethrin and PBO content in the tested Olyset® Net and Olyset® Plus did not decrease significantly (Table 3).

## Discussion

### Summary of the Experiments

The rationale behind combining PBO with a pyrethroid relies on the action of PBO as a metabolic enzyme inhibitor, which might enhance the efficacy of pyrethroids against mosquitoes bearing metabolic-based resistance mechanisms. Moreover it has been demonstrated that it might act as an adjuvant through its effect on enhanced cuticular penetration of deltamethrin [32]. Development of a net incorporating a pyrethroid with a synergist is promising against pyrethroid resistant malaria vectors. This study revealed that Olyset® Plus has better efficacy than the standard Olyset® Net under both laboratory and semi-field conditions. This study demonstrated the benefit of incorporating PBO and permethrin together in a long-lasting insecticidal net for malaria vector control.

### Regeneration Time and Washing Resistance

In this study, the regeneration time of the Olyset® Plus was determined at 2 days after 3 consecutive washes on same day when the efficacy reached a plateau. It is interesting to note that two regeneration patterns might be observed among the LLINs reviewed by WHOPES: 1) both KD effect and mortality reached the initial levels of efficacy after washing; 2) the KD effect reached the initial efficacy and the mortality reached a plateau. Olyset® Plus after washing induced a KD affect as good as the initial one and the mortality reached a plateau 2 days after the last wash. The permethrin content decreased from 816.5±2.0 mg/m^2^ to 685.9±3.5 mg/m^2^, indicating that the proportion of the active ingredient bio-available on the net surface was depleted by the washes. This contrasted with the standard Olyset® Net of which there was no significant decrease of permethrin content (919.7±1.7 mg/m^2^ before vs 907.4±5.7 mg/m^2^ after the 3 washes) as previously reported [33]. A different technology used to incorporate permethrin into the Olyset® Plus fibres probably setting a different bleeding rate of the active ingredient clearly improved the bio-availability of the insecticide and hence the insecticidal activity of the net.

The same trend was observed with the wash resistance study. After washing, the permethrin content decreased from 816 mg/m^2^ to 519 mg/m^2^ and the PBO content from 384 mg/m^2^ to 172 mg/m^2^. The retention rates were respectively 64% for permethrin and 45% for PBO after 25 washes. These chemical contents of Olyset® Plus led to a decrease of the induced mortality (from 100% to 16%) whereas the KD effect was still above 95% against susceptible *An. gambiae.* This contrasts with Olyset® Net for which no significant decrease in permethrin content after washing has been reported [34].

### Efficacy Against Multi-resistant Mosquito Strains

The release-recapture experiments conducted in Benin and Cameroun aimed at evaluating the efficacy of Olyset® Plus against wild *An. gambiae* populations with different pyrethroid resistance mechanisms [30], [35]–[37]. In the north of Cameroun, resistance to pyrethroids relies mainly on metabolic mechanisms [35] whereas the *An. gambiae* population from Benin is sharing the west-African *Kdr* mutation at a very high frequency (0.85 to 0.98) in addition to metabolic resistance mechanisms [30]. Olyset® Plus induced comparable exophily and blood-feeding inhibition among the resistant populations from Benin and Cameroun than the Olyset® Net. In contrast, Olyset® Plus performed better than Olyset Net® in terms of killing effect against both the pyrethroid resistant populations. These results confirmed the good efficacy of Olyset® Plus regardless the presence or absence of the *Kdr* mutation. Nevertheless, the mortality was slightly (but not significantly) lower in Cotonou than in Pitoa. This decrease of mortality rate may be due to the *Kdr* mutation in the genetic backgrounds of the *An. gambiae* s.l. populations from the study sites, although the impact of *Kdr* on the phenotype of resistance to LLINs should be further elucidated [2].

One should note that during the release-recapture experiment we compared the Olyset® Plus with a positive control made by the same technology and with the same fabrics but without PBO, called Olyset® Plus without PBO. Indeed Olyset® Plus (washed 3 times) induced significantly higher mortality than Olyset® Plus without PBO against both the resistant strains from Benin (59% *vs* 18% mortality) and Cameroun (69 *vs* 52% mortality) hence demonstrating a significant additional effect of PBO. A better efficacy of combination LLIN containing deltamethrin and PBO on the roof panel compared to deltamethrin alone has already been reported [16], [18], [19], [38]. However, a different technology, fabrics and deltamethrin contents were used in manufacturing the combination LLIN and the deltamethrin-treated LLIN that rendered difficult the interpretation of the results. With our specific study design and using Olyset® Plus without PBO as a positive control, we succeeded to show an improved bio-availability and benefits of using PBO and permethrin in the LLIN fibres.

### Phase II Studies

The experimental hut study (Phase II) was conducted in an area of moderate pyrethroid resistance where *An. gambiae* populations exhibit both target site mutations and metabolic mechanisms [30]. Higher efficacy of Olyset® Plus was confirmed compared to the standard Olyset Net® in terms of insecticidal activity but Olyset® Plus induced lower exophily than Olyset Net®. The mortality rates induced by Olyset® Plus and Olyset Net® declined significantly after 20 washes (67% to 36%, and 81% to 42% respectively). More interesting is the impact of 20 washes on the personal protection (i.e. BFI). Before washing, Olyset® Plus and Olyset® Net inhibited blood-feeding equally, but after 20 washes Olyset® Plus conferred a higher protection to the person sleeping under than the Olyset® Net. This confirmed previous results obtained with the combination LLIN PermaNet 3.0 showing that washing attenuated personal protection but the direct protective efficacy of the combination product proved to be more durable [16], [20].

Results for Culicidae mosquitoes mirrored that of *An. gambiae s.l.*, except for the proportions of mosquitoes exiting to the verandas. There were no significant differences in entry rates (deterrence) and exit rates (exophily) of Culicidae between treatments and the control huts. Blood-feeding inhibition rates for all treatments were high (>93%) compared with the control; there was no significant difference between the treatments (Table 4) most probably because of the susceptibility and heterogeneity of the mosquito population considered. All treatments caused high mortality of Culicidae mosquitoes (85–96%) relative to the untreated net (2%). Such a protective efficacy against nuisance causing mosquitoes is a positive factor as the personal protection against general blood sucking insects might be a key factor of acceptability and proper use of the LLINs.

The contents of permethrin and PBO in unwashed Olyset® Plus samples tested in phase I and II studies complied with their target doses of 20±5 g AI/kg and 10±2.5 g PBO/kg, respectively. The between-net variation of the permethrin and PBO contents was within the limits specified by the WHO guidelines indicating a good homogeneity. The bioassays and chemical analysis from phase I wash resistance studies showed an increased release rate of permethrin and a shorter regeneration time of 2 days of Olyset® Plus compared with Olyset® Net emphasizing the difference in the long-lasting technology that allow better availability of the permethrin in the Olyset® Plus.

Beyond the intrinsic efficacy of Olyset® Plus, this study emphasizes the potential benefit of incorporating an insecticide active ingredient and a synergist into a net to better control malaria vectors and/or prevent or delay the development of insecticide resistance. This approach might not only include compounds with synergistic effect on insect nervous system (i.e. two different neurotoxic insecticides [39]–[41] or one insecticide and one repellent [42]–[45]) but also compounds acting on different physiological targets such as entomopathogenic fungi [46] or insect growth regulators [47].

Nevertheless in the race to limit malaria transmission, it is also crucial to devise different tools other than LLINs and indoor residual spraying) targeting outdoor or diurnal biting vectors as more and more evidences of mosquito behavioral changes might render all of these insecticidal combinations useless [13], [48].

## Conclusions and Perspectives

The present study showed evidence for the benefit of combining a synergist, PBO, to a pyrethroid insecticide into a mosquito net. The new LLIN, Olyset® Plus, showed significantly better performance against multiple resistant populations of *An. gambiae* than the standard Olyset Net®. These encouraging results need to be complemented by a large-scale field trial to assess the durability and acceptability of this new vector control tool for malaria vector control.
