# Correction: Efficacy of Olyset® Plus, a New Long-Lasting Insecticidal Net Incorporating Permethrin and Piperonil-Butoxide against Multi-Resistant Malaria Vectors

**DOI:** 10.1371/annotation/bed4305a-d665-4150-a682-a20d9cf9b79f

**Published:** 2013-10-10

**Authors:** Cédric Pennetier, Aziz Bouraima, Fabrice Chandre, Michael Piameu, Josiane Etang, Marie Rossignol, Ibrahim Sidick, Barnabas Zogo, Marie-Noëlle Lacroix, Rajpal Yadav, Olivier Pigeon, Vincent Corbel1

There was an error in the title.

The correct version of the title is: Efficacy of Olyset® Plus, a New Long-Lasting Insecticidal Net Incorporating Permethrin and Piperonyl-Butoxide against Multi-Resistant Malaria Vectors

The correct citation is: Pennetier C, Bouraima A, Chandre F, Piameu M, Etang J, et al. (2013) Efficacy of Olyset® Plus, a New Long-Lasting Insecticidal Net Incorporating Permethrin and Piperonyl-Butoxide against Multi-Resistant Malaria Vectors. PLoS ONE 8(10): e75134. doi:10.1371/journal.pone.0075134 

